# Poly[[μ_2_-aqua-μ_3_-(4-carb­oxy-2-propyl-1*H*-imidazole-5-carboxyl­ato-κ^4^ *N* ^3^,*O* ^4^:*O* ^4^:*O* ^5^)-sodium] hemihydrate]

**DOI:** 10.1107/S1600536811007732

**Published:** 2011-03-09

**Authors:** Zhong-Jing Huang, Jin-Niu Tang, Zhi-Rong Luo, Dai-Yin Wang, Huan Wei

**Affiliations:** a Department of Chemistry, Guangxi University for Nationalities, Nanning 530006, People’s Republic of China

## Abstract

In the title compound, {[Na(C_8_H_9_N_2_O_4_)(H_2_O)]·0.5H_2_O}_*n*_, the Na^+^ ion is coordinated by two bridging water mol­ecules, one N atom and three O atoms from three 4-carb­oxy-2-propyl-1*H*-imidazole-5-carboxyl­ate (H_2_pimdc) ligands. Adjacent Na^+^ ions are linked alternately by two water O atoms and two carb­oxy O atoms into a chain along [001]. These chains are connected through the coordination of the carboxyl­ate O atoms to the Na^+^ ions, forming a three-dimensional structure. An intra­molecular O—H⋯O hydrogen bond and inter­molecular N—H⋯O and O—H⋯O hydrogen bonds are present in the crystal structure.

## Related literature

For the properties and biological activity of imidazole-4,5-dicarb­oxy­lic acid and its derivatives, see: Baures (1999 ▶); Bogdanova *et al.* (1992 ▶); Borodkin *et al.* (1984 ▶); Reichardt *et al.* (1992 ▶); Su *et al.* (2001 ▶).
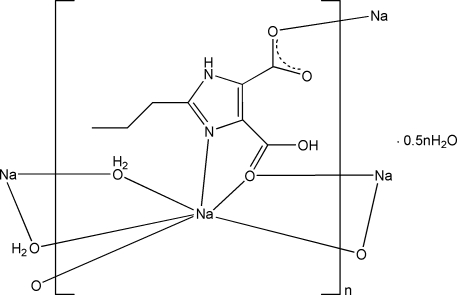


## Experimental

### 

#### Crystal data

[Na(C_8_H_9_N_2_O_4_)(H_2_O)]·0.5H_2_O
*M* *_r_* = 247.18
Monoclinic, 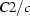

*a* = 15.406 (4) Å
*b* = 15.478 (4) Å
*c* = 10.734 (3) Å
β = 118.364 (3)°
*V* = 2252.4 (9) Å^3^
*Z* = 8
Mo *K*α radiation
μ = 0.15 mm^−1^
*T* = 296 K
0.52 × 0.47 × 0.44 mm

#### Data collection

Bruker APEX CCD diffractometer
Absorption correction: multi-scan (*SADABS*; Sheldrick, 1996 ▶) *T*_min_ = 0.528, *T*_max_ = 0.562
5971 measured reflections
1986 independent reflections
1669 reflections with *I* > 2σ(*I*)
*R*_int_ = 0.020

#### Refinement

*R*[*F* ^2^ > 2σ(*F* ^2^)] = 0.034
*wR*(*F* ^2^) = 0.106
*S* = 1.10
1986 reflections
152 parameters
H-atom parameters constrained
Δρ_max_ = 0.23 e Å^−3^
Δρ_min_ = −0.21 e Å^−3^

### 

Data collection: *SMART* (Bruker, 2007 ▶); cell refinement: *SAINT* (Bruker, 2007 ▶); data reduction: *SAINT*; program(s) used to solve structure: *SHELXS97* (Sheldrick, 2008 ▶); program(s) used to refine structure: *SHELXL97* (Sheldrick, 2008 ▶); molecular graphics: *DIAMOND* (Brandenburg, 1999 ▶); software used to prepare material for publication: *SHELXTL* (Sheldrick, 2008 ▶).

## Supplementary Material

Crystal structure: contains datablocks I, global. DOI: 10.1107/S1600536811007732/hy2402sup1.cif

Structure factors: contains datablocks I. DOI: 10.1107/S1600536811007732/hy2402Isup2.hkl

Additional supplementary materials:  crystallographic information; 3D view; checkCIF report

## Figures and Tables

**Table 1:** Selected bond lengths (Å)

Na1—O1	2.3658 (15)
Na1—O1^i^	2.3644 (14)
Na1—O3^ii^	2.5550 (15)
Na1—O5	2.4011 (15)
Na1—O5^iii^	2.3818 (16)
Na1—N1	2.4848 (16)

Symmetry codes: (i) 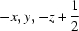; (ii) 
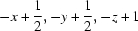
; (iii) 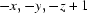.

**Table 2:** Hydrogen-bond geometry (Å, °)

*D*—H⋯*A*	*D*—H	H⋯*A*	*D*⋯*A*	*D*—H⋯*A*
N2—H2*A*⋯O3^iv^	0.86	2.00	2.8384 (18)	164
O2—H2⋯O4	0.82	1.64	2.4603 (18)	178
O5—H5*B*⋯O2^i^	0.91	2.07	2.9493 (18)	164
O5—H5*A*⋯O6	0.86	1.97	2.8234 (19)	169
O6—H6*C*⋯O4^ii^	0.88	2.03	2.8835 (16)	163

Symmetry codes: (i) 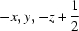; (ii) 
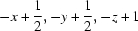
; (iv) 
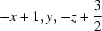
.

**Fig. 1.**
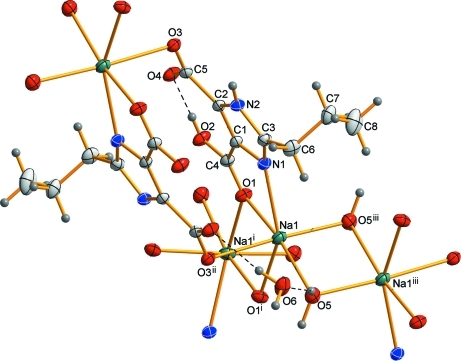
The structure of the title compound. Displacement ellipsoids are drawn at the 30% probability level. Dashed lines denote hydrogen bonds. [Symmetry codes: (i) -x, y, 1/2-z; (ii) 1/2-x, 1/2-y, 1-z; (iii) -x, -y, 1-z.]

**Fig. 2.**
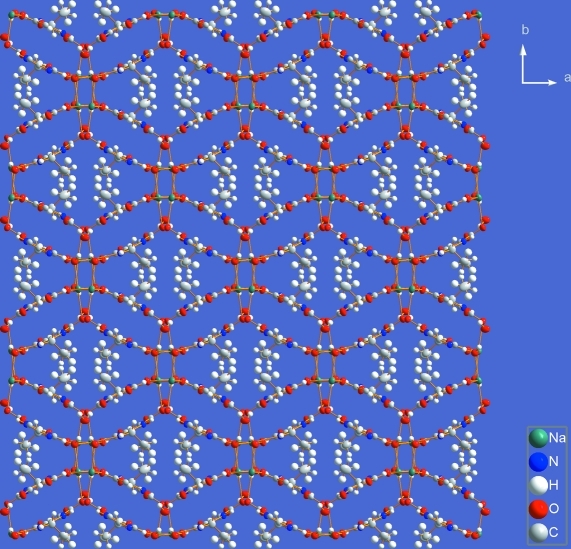
Crystal packing of the title compound viewed along the c axis.

## supplementary crystallographic information

### Comment

1*H*-Imidazole and its derivatives are a kind of excellent supramolecular synthons. The N atoms of imidazole can coordinate to metal ions in monodentate or bidentate mode (Su *et al.*, 2001). Imidazole-4,5-dicarboxylic acid and derivatives have been studied in terms of their physical properties as well as for their diverse biological activities (Bogdanova *et al.*, 1992; Borodkin *et al.*, 1984; Reichardt *et al.*, 1992), including the use of imidazole-4,5-dicarboxylic acid amides in the development of human immunodeficiency virus (HIV-1) protease inhibitors (Baures, 1999). Here we present the synthesis and structure of the title complex derived from 2-propyl-1*H*-imidazole-4,5-dicarboxylic acid. The distinct binding modes of the ligands as well as the coordination preferences of the metal ion are discussed.

In the title compound, the 2-propyl-4-carboxy-1*H*-imidazole-5-carboxylate (H_2_pimdc) ligand is bonded to Na ions in a µ_3_-mode. The coordination of Na ion is achieved by two water molecules, one N atom and three O atoms from three H_2_pimdc ligands (Fig .1, Table 1). Two O1 atoms of the H_2_pimdc ligand bridge two Na atoms, forming a parallelogram and two O5 atoms of the water molecules form another parallelogram with two Na atoms, leading to a chain along [0 0 1]. The chains are connected through the coordination of the carboxylate O3 atoms to the Na ions, forming a three-dimensional structure (Fig. 2). The propyl group becomes an ornament in the coordination network. The crystal structure is stabilized by intramolecular O—H···O hydrogen bond and intermolecular N—H···O and O—H···O hydrogen bonds (Table 2).

### Experimental

An ethanol solution (5 ml) containing 2-propyl-1H-imidazole-4,5-dicarboxylic acid (1 mmol, 0.198 g) was added dropwise to a water solution (10 ml) containing NaOH (0.5 mmol, 0.040 g). After stirring for 6 h, the solution was filtered. The filtered solution were evaporated for several days in air and colorless block-shaped crystals suitable for single-crystal X-ray diffraction were obtained (yield: 80% based on the ligand).

### Refinement

H atoms except those of water molecules were positioned geometrically and refined using a riding model, with C—H = 0.97 (CH_2_) and 0.96 (CH_3_), N—H = 0.86 and O—H = 0.82 Å and with *U*_iso_(H) = 1.2(1.5 for methyl)*U*_eq_(C,N,O). H atoms of water molecules were located in a difference Fourier map and refined as riding atoms, with *U*_iso_(H) = 1.2*U*_eq_(O).

### Crystal data

**Table:**

[Na(C_8_H_9_N_2_O_4_)(H_2_O)]·0.5H_2_O	*F*(000) = 1032
*M**_r_* = 247.18	*D*_x_ = 1.458 Mg m^−^^3^
Monoclinic, *C*2/*c*	Mo *K*α radiation, λ = 0.71073 Å
Hall symbol: -C 2yc	Cell parameters from 2142 reflections
*a* = 15.406 (4) Å	θ = 2.4–26.8°
*b* = 15.478 (4) Å	µ = 0.15 mm^−^^1^
*c* = 10.734 (3) Å	*T* = 296 K
β = 118.364 (3)°	Block, colorless
*V* = 2252.4 (9) Å^3^	0.52 × 0.47 × 0.44 mm
*Z* = 8	

### Data collection

**Table:**

Bruker APEX CCD diffractometer	1986 independent reflections
Radiation source: fine-focus sealed tube	1669 reflections with *I* > 2σ(*I*)
graphite	*R*_int_ = 0.020
φ and ω scans	θ_max_ = 25.0°, θ_min_ = 2.0°
Absorption correction: multi-scan (*SADABS*; Sheldrick, 1996)	*h* = −18→17
*T*_min_ = 0.528, *T*_max_ = 0.562	*k* = −18→17
5971 measured reflections	*l* = −11→12

### Refinement

**Table:**

Refinement on *F*^2^	Primary atom site location: structure-invariant direct methods
Least-squares matrix: full	Secondary atom site location: difference Fourier map
*R*[*F*^2^ > 2σ(*F*^2^)] = 0.034	Hydrogen site location: inferred from neighbouring sites
*wR*(*F*^2^) = 0.106	H-atom parameters constrained
*S* = 1.10	*w* = 1/[σ^2^(*F*_o_^2^) + (0.0609*P*)^2^ + 0.6897*P*] where *P* = (*F*_o_^2^ + 2*F*_c_^2^)/3
1986 reflections	(Δ/σ)_max_ < 0.001
152 parameters	Δρ_max_ = 0.23 e Å^−^^3^
0 restraints	Δρ_min_ = −0.21 e Å^−^^3^

### Fractional atomic coordinates and isotropic or equivalent isotropic displacement parameters (Å^2^)

**Table:**

	*x*	*y*	*z*	*U*_iso_*/*U*_eq_	
O3	0.48386 (8)	0.25613 (8)	0.57288 (12)	0.0379 (3)	
C5	0.41043 (11)	0.21569 (11)	0.48692 (17)	0.0310 (4)	
Na1	0.04274 (5)	0.08063 (4)	0.43441 (7)	0.0385 (2)	
O1	0.10965 (9)	0.08373 (8)	0.27653 (13)	0.0411 (3)	
O2	0.22662 (9)	0.13103 (9)	0.23128 (12)	0.0416 (3)	
H2	0.2806	0.1544	0.2746	0.062*	
O4	0.38682 (9)	0.20490 (9)	0.35643 (12)	0.0417 (3)	
N2	0.36555 (10)	0.18188 (9)	0.67516 (14)	0.0321 (3)	
H2A	0.4177	0.2046	0.7425	0.039*	
O5	−0.06348 (10)	0.07199 (8)	0.54055 (14)	0.0437 (3)	
H5A	−0.0517	0.1150	0.5978	0.052*	
H5B	−0.1209	0.0894	0.4662	0.052*	
N1	0.22111 (10)	0.11704 (9)	0.56488 (14)	0.0329 (3)	
C2	0.34437 (11)	0.17694 (10)	0.53667 (16)	0.0290 (4)	
C4	0.19168 (12)	0.11485 (11)	0.31776 (17)	0.0313 (4)	
C1	0.25389 (11)	0.13611 (10)	0.46930 (16)	0.0292 (4)	
C3	0.29110 (12)	0.14524 (11)	0.68890 (18)	0.0338 (4)	
C6	0.29259 (14)	0.13640 (14)	0.82798 (19)	0.0454 (5)	
H6A	0.2276	0.1191	0.8121	0.054*	
H6B	0.3074	0.1922	0.8748	0.054*	
C7	0.36813 (17)	0.07062 (14)	0.9247 (2)	0.0525 (5)	
H7A	0.3512	0.0141	0.8804	0.063*	
H7B	0.4327	0.0861	0.9367	0.063*	
C8	0.3727 (2)	0.06594 (17)	1.0677 (2)	0.0757 (8)	
H8A	0.3924	0.1210	1.1138	0.113*	
H8B	0.4198	0.0228	1.1242	0.113*	
H8C	0.3089	0.0510	1.0564	0.113*	
O6	0.0000	0.20200 (12)	0.7500	0.0512 (5)	
H6C	0.0310	0.2400	0.7245	0.061*	

### Atomic displacement parameters (Å^2^)

**Table:**

	*U*^11^	*U*^22^	*U*^33^	*U*^12^	*U*^13^	*U*^23^
O3	0.0294 (6)	0.0443 (7)	0.0345 (7)	−0.0048 (5)	0.0108 (5)	0.0028 (5)
C5	0.0256 (8)	0.0338 (9)	0.0317 (9)	0.0040 (7)	0.0121 (7)	0.0032 (7)
Na1	0.0328 (4)	0.0462 (4)	0.0354 (4)	−0.0046 (3)	0.0154 (3)	0.0024 (3)
O1	0.0309 (7)	0.0564 (8)	0.0319 (7)	−0.0082 (6)	0.0115 (5)	−0.0032 (5)
O2	0.0347 (7)	0.0600 (8)	0.0289 (6)	−0.0111 (6)	0.0142 (5)	−0.0073 (6)
O4	0.0391 (7)	0.0574 (8)	0.0332 (7)	−0.0083 (6)	0.0209 (6)	−0.0025 (5)
N2	0.0269 (7)	0.0400 (8)	0.0254 (7)	−0.0013 (6)	0.0091 (5)	−0.0007 (6)
O5	0.0472 (8)	0.0453 (8)	0.0372 (7)	0.0025 (6)	0.0190 (6)	0.0020 (5)
N1	0.0298 (7)	0.0401 (8)	0.0291 (7)	−0.0001 (6)	0.0142 (6)	0.0024 (6)
C2	0.0288 (8)	0.0305 (8)	0.0266 (8)	0.0032 (7)	0.0122 (7)	0.0004 (6)
C4	0.0299 (9)	0.0346 (9)	0.0280 (9)	0.0012 (7)	0.0126 (7)	0.0000 (7)
C1	0.0270 (8)	0.0319 (9)	0.0282 (9)	0.0030 (6)	0.0127 (7)	0.0017 (6)
C3	0.0299 (9)	0.0408 (10)	0.0294 (9)	0.0031 (7)	0.0131 (7)	0.0034 (7)
C6	0.0457 (11)	0.0611 (12)	0.0319 (10)	−0.0014 (9)	0.0205 (8)	0.0018 (9)
C7	0.0587 (13)	0.0574 (13)	0.0383 (11)	−0.0110 (10)	0.0205 (9)	0.0054 (9)
C8	0.104 (2)	0.0765 (17)	0.0368 (12)	−0.0225 (15)	0.0254 (13)	0.0041 (11)
O6	0.0693 (13)	0.0439 (11)	0.0553 (12)	0.000	0.0417 (11)	0.000

### Geometric parameters (Å, °)

**Table:**

O3—C5	1.237 (2)	O5—H5A	0.8645
C5—O4	1.280 (2)	O5—H5B	0.9066
C5—C2	1.482 (2)	N1—C3	1.327 (2)
Na1—O1	2.3658 (15)	N1—C1	1.374 (2)
Na1—O1^i^	2.3644 (14)	C2—C1	1.381 (2)
Na1—O3^ii^	2.5550 (15)	C4—C1	1.480 (2)
Na1—O5	2.4011 (15)	C3—C6	1.488 (2)
Na1—O5^iii^	2.3818 (16)	C6—C7	1.524 (3)
Na1—N1	2.4848 (16)	C6—H6A	0.9700
Na1—Na1^iii^	3.4217 (14)	C6—H6B	0.9700
Na1—Na1^i^	3.5291 (16)	C7—C8	1.504 (3)
O1—C4	1.222 (2)	C7—H7A	0.9700
O2—C4	1.300 (2)	C7—H7B	0.9700
O2—H2	0.8200	C8—H8A	0.9600
N2—C3	1.349 (2)	C8—H8B	0.9600
N2—C2	1.364 (2)	C8—H8C	0.9600
N2—H2A	0.8600	O6—H6C	0.8790
			
C5—O3—Na1^ii^	113.64 (10)	Na1^iii^—O5—Na1	91.35 (5)
O3—C5—O4	124.46 (15)	Na1^iii^—O5—H5A	135.4
O3—C5—C2	118.49 (15)	Na1—O5—H5A	109.5
O4—C5—C2	117.05 (14)	Na1^iii^—O5—H5B	114.6
O1^i^—Na1—O1	83.45 (5)	Na1—O5—H5B	99.5
O1^i^—Na1—O5^iii^	98.46 (5)	H5A—O5—H5B	100.6
O1—Na1—O5^iii^	91.16 (5)	C3—N1—C1	105.47 (14)
O1^i^—Na1—O5	82.24 (5)	C3—N1—Na1	142.55 (12)
O1—Na1—O5	165.50 (5)	C1—N1—Na1	109.17 (10)
O5^iii^—Na1—O5	88.65 (5)	N2—C2—C1	104.80 (14)
O1^i^—Na1—N1	149.80 (6)	N2—C2—C5	121.35 (14)
O1—Na1—N1	69.97 (5)	C1—C2—C5	133.75 (15)
O5^iii^—Na1—N1	96.21 (5)	O1—C4—O2	121.69 (15)
O5—Na1—N1	124.46 (6)	O1—C4—C1	120.25 (15)
O1^i^—Na1—O3^ii^	83.03 (5)	O2—C4—C1	118.06 (14)
O1—Na1—O3^ii^	94.36 (5)	N1—C1—C2	110.18 (14)
O5^iii^—Na1—O3^ii^	174.41 (5)	N1—C1—C4	119.81 (14)
O5—Na1—O3^ii^	86.22 (5)	C2—C1—C4	129.97 (15)
N1—Na1—O3^ii^	84.91 (5)	N1—C3—N2	110.88 (15)
O1^i^—Na1—Na1^iii^	90.44 (4)	N1—C3—C6	126.43 (16)
O1—Na1—Na1^iii^	133.90 (5)	N2—C3—C6	122.65 (15)
O5^iii^—Na1—Na1^iii^	44.55 (4)	C3—C6—C7	112.82 (16)
O5—Na1—Na1^iii^	44.10 (4)	C3—C6—H6A	109.0
N1—Na1—Na1^iii^	118.18 (5)	C7—C6—H6A	109.0
O3^ii^—Na1—Na1^iii^	130.28 (5)	C3—C6—H6B	109.0
O1^i^—Na1—Na1^i^	41.76 (4)	C7—C6—H6B	109.0
O1—Na1—Na1^i^	41.73 (3)	H6A—C6—H6B	107.8
O5^iii^—Na1—Na1^i^	94.88 (4)	C8—C7—C6	112.2 (2)
O5—Na1—Na1^i^	123.85 (5)	C8—C7—H7A	109.2
N1—Na1—Na1^i^	110.83 (4)	C6—C7—H7A	109.2
O3^ii^—Na1—Na1^i^	89.81 (3)	C8—C7—H7B	109.2
Na1^iii^—Na1—Na1^i^	116.76 (3)	C6—C7—H7B	109.2
C4—O1—Na1^i^	137.76 (11)	H7A—C7—H7B	107.9
C4—O1—Na1	118.44 (11)	C7—C8—H8A	109.5
Na1^i^—O1—Na1	96.50 (5)	C7—C8—H8B	109.5
C4—O2—H2	109.5	H8A—C8—H8B	109.5
C3—N2—C2	108.66 (14)	C7—C8—H8C	109.5
C3—N2—H2A	125.7	H8A—C8—H8C	109.5
C2—N2—H2A	125.7	H8B—C8—H8C	109.5
			
Na1^ii^—O3—C5—O4	83.19 (17)	Na1^iii^—Na1—N1—C1	142.32 (10)
Na1^ii^—O3—C5—C2	−96.58 (14)	Na1^i^—Na1—N1—C1	3.70 (11)
O1^i^—Na1—O1—C4	153.03 (11)	C3—N2—C2—C1	0.27 (17)
O5^iii^—Na1—O1—C4	−108.59 (13)	C3—N2—C2—C5	177.03 (14)
O5—Na1—O1—C4	162.3 (2)	O3—C5—C2—N2	−3.0 (2)
N1—Na1—O1—C4	−12.40 (12)	O4—C5—C2—N2	177.24 (15)
O3^ii^—Na1—O1—C4	70.56 (13)	O3—C5—C2—C1	172.70 (17)
Na1^iii^—Na1—O1—C4	−122.69 (12)	O4—C5—C2—C1	−7.1 (3)
Na1^i^—Na1—O1—C4	155.37 (15)	Na1^i^—O1—C4—O2	−27.1 (3)
O1^i^—Na1—O1—Na1^i^	−2.35 (7)	Na1—O1—C4—O2	−169.10 (12)
O5^iii^—Na1—O1—Na1^i^	96.04 (5)	Na1^i^—O1—C4—C1	152.23 (13)
O5—Na1—O1—Na1^i^	7.0 (2)	Na1—O1—C4—C1	10.2 (2)
N1—Na1—O1—Na1^i^	−167.77 (6)	C3—N1—C1—C2	−0.50 (19)
O3^ii^—Na1—O1—Na1^i^	−84.81 (5)	Na1—N1—C1—C2	165.02 (11)
Na1^iii^—Na1—O1—Na1^i^	81.93 (7)	C3—N1—C1—C4	−178.63 (15)
O1^i^—Na1—O5—Na1^iii^	98.73 (5)	Na1—N1—C1—C4	−13.10 (18)
O1—Na1—O5—Na1^iii^	89.4 (2)	N2—C2—C1—N1	0.15 (18)
O5^iii^—Na1—O5—Na1^iii^	0.0	C5—C2—C1—N1	−176.03 (16)
N1—Na1—O5—Na1^iii^	−96.60 (7)	N2—C2—C1—C4	178.02 (16)
O3^ii^—Na1—O5—Na1^iii^	−177.79 (5)	C5—C2—C1—C4	1.8 (3)
Na1^i^—Na1—O5—Na1^iii^	94.97 (4)	O1—C4—C1—N1	3.0 (2)
O1^i^—Na1—N1—C3	139.23 (18)	O2—C4—C1—N1	−177.59 (15)
O1—Na1—N1—C3	169.0 (2)	O1—C4—C1—C2	−174.66 (17)
O5^iii^—Na1—N1—C3	−102.0 (2)	O2—C4—C1—C2	4.7 (3)
O5—Na1—N1—C3	−9.4 (2)	C1—N1—C3—N2	0.68 (19)
O3^ii^—Na1—N1—C3	72.5 (2)	Na1—N1—C3—N2	−156.48 (14)
Na1^iii^—Na1—N1—C3	−61.0 (2)	C1—N1—C3—C6	−177.21 (17)
Na1^i^—Na1—N1—C3	160.36 (18)	Na1—N1—C3—C6	25.6 (3)
O1^i^—Na1—N1—C1	−17.43 (17)	C2—N2—C3—N1	−0.61 (19)
O1—Na1—N1—C1	12.38 (10)	C2—N2—C3—C6	177.38 (16)
O5^iii^—Na1—N1—C1	101.34 (11)	N1—C3—C6—C7	107.8 (2)
O5—Na1—N1—C1	−166.03 (10)	N2—C3—C6—C7	−69.8 (2)
O3^ii^—Na1—N1—C1	−84.16 (11)	C3—C6—C7—C8	176.85 (18)

Symmetry codes: (i) −*x*, *y*, −*z*+1/2; (ii) −*x*+1/2, −*y*+1/2, −*z*+1; (iii) −*x*, −*y*, −*z*+1.

### Hydrogen-bond geometry (Å, °)

**Table:**

*D*—H···*A*	*D*—H	H···*A*	*D*···*A*	*D*—H···*A*
N2—H2A···O3^iv^	0.86	2.00	2.8384 (18)	164
O2—H2···O4	0.82	1.64	2.4603 (18)	178
O5—H5B···O2^i^	0.91	2.07	2.9493 (18)	164
O5—H5A···O6	0.86	1.97	2.8234 (19)	169
O6—H6C···O4^ii^	0.88	2.03	2.8835 (16)	163

Symmetry codes: (iv) −*x*+1, *y*, −*z*+3/2; (i) −*x*, *y*, −*z*+1/2; (ii) −*x*+1/2, −*y*+1/2, −*z*+1.
